# Descriptor Free QSAR Modeling Using Deep Learning With Long Short-Term Memory Neural Networks

**DOI:** 10.3389/frai.2019.00017

**Published:** 2019-09-06

**Authors:** Suman K. Chakravarti, Sai Radha Mani Alla

**Affiliations:** MultiCASE Inc., Beachwood, OH, United States

**Keywords:** QSAR (quantitative structure-activity relationships), machine learning, mutagenicity, big data, LSTM (long short term memory networks), RNN (recurrent neural network), malaria, hepatitis (C) virus

## Abstract

Current practice of building QSAR models usually involves computing a set of descriptors for the training set compounds, applying a descriptor selection algorithm and finally using a statistical fitting method to build the model. In this study, we explored the prospects of building good quality interpretable QSARs for big and diverse datasets, without using any pre-calculated descriptors. We have used different forms of Long Short-Term Memory (LSTM) neural networks to achieve this, trained directly using either traditional SMILES codes or a new linear molecular notation developed as part of this work. Three endpoints were modeled: Ames mutagenicity, inhibition of *P. falciparum* Dd2 and inhibition of Hepatitis C Virus, with training sets ranging from 7,866 to 31,919 compounds. To boost the interpretability of the prediction results, attention-based machine learning mechanism, jointly with a bidirectional LSTM was used to detect structural alerts for the mutagenicity data set. Traditional fragment descriptor-based models were used for comparison. As per the results of the external and cross-validation experiments, overall prediction accuracies of the LSTM models were close to the fragment-based models. However, LSTM models were superior in predicting test chemicals that are dissimilar to the training set compounds, a coveted quality of QSAR models in real world applications. In summary, it is possible to build QSAR models using LSTMs without using pre-computed traditional descriptors, and models are far from being “black box.” We wish that this study will be helpful in bringing large, descriptor-less QSARs to mainstream use.

## Introduction

Quantitative structure-activity relationship (QSAR) based approaches have proven to be very valuable in predicting physicochemical properties, biological activity, toxicity, chemical reactivity, and metabolism of chemical compounds (Hansch and Fujita, 1964; Hansch and Leo, 1979; Zhu et al., 2005; Cherkasov et al., 2014; Neves et al., 2018). QSAR approaches are increasingly being accepted within regulatory decision-making process as an alternative to animal tests for toxicity screening of chemicals [(M7(R1), 2018)].

From the beginning, QSAR is largely a process of relating a set of predictor variables (*X*) to the response variable (*Y*) (Hansch and Fujita, 1964; Hansch and Leo, 1979). A vast amount of research efforts has been spent on the methods for linking *X* and *Y*, and on the predictors or molecular descriptors themselves. Physicochemical, graph theoretical, or mathematical descriptors have helped the QSAR field to thrive (Karelson et al., 1996). However, descriptors are also becoming a liability (Ghasemi et al., 2018) because many of them are hard to explain in terms of how they are related to the target activity, they are indirect representations of chemical structures, introduce human bias, and a significant software framework is needed to compute the descriptors themselves. It is getting increasingly difficult to find descriptors to build QSARs from diverse and large datasets of bio-assays. Fragment-based descriptors solve some of these problems, as fragments are direct representations of chemical structures and easier to generate (Sutherland et al., 2008; Salum and Andricopulo, 2010). However, selecting a few relevant fragments from a large pool is a tough challenge, and to make the situation worse, current fragment representations produce large and sparse X matrices (Chakravarti, 2018).

With these issues in mind, our objective was to explore the possibility of completely eliminating molecular descriptors for building QSARs, primarily for large and diverse datasets. In this study, we have used the deep learning abilities of long short-term memory networks (LSTMs), to learn directly from SMILES code of molecular structures. The methodology was applied to both toxicity and pharmacological end points, using big training sets ranging from 7,866 to 31,919 compounds. Use of deep learning techniques in various areas of cheminformatics is increasing rapidly in recent years (Goh et al., 2017). Recently Fooshee et al. (2018) reported deep learning of chemical reactions by training LSTMs directly using SMILES strings. Efforts of descriptor-less QSARs have also been published (Alessandro et al., 2013; Coley et al., 2017), however, they were based on smaller data sets, required significant structure preprocessing software framework and limited in scope. Toropov et al. (2008, 2009, 2013) used so called “optimal descriptors” computed directly from SMILES and InChI codes to model octanol-water partition coefficients of Platinum complexes and vitamins and water solubility. Cao et al. (2012) used fragments of SMILES code as descriptors to create SVM based models for predicting various toxic properties. However, descriptors are still involved in both of these studies, and the training sets are small, <100 and 1,000 for Toropov et al. and Cao et al., respectively.

LSTM networks are a type of recurrent neural network (RNN) architecture used for modeling sequence data (Hochreiter and Schmidhuber, 1997; Greff et al., 2015). Jastrzebski et al. (2016) directly used SMILES via convolutional and recurrent neural networks for building predictive models. Although this work is conceptually quite similar, the publication lacks sufficient details and the datasets are fairly small (mean size 3,000). They reported identifying biologically relevant substructures using data from convolutional filters but did not try the same from the recurrent neural networks. A different type of approach reported by Winter et al. (2019) and Gómez-Bombarelli et al. (2018), who used conversion between molecular linear representations to learn continuous latent vector forms of molecules and consequently used them for QSAR or designing molecules. For the present work, LSTMs are particularly advantageous because they can work with training data that have input of different lengths in different examples, can take advantage of same features present at different positions in the input sequence data and therefore, learn better. This is in contrast with a traditional QSAR, in which the *X* descriptor matrix have to be of fixed number of columns and column position of every descriptor should remain fixed.

We have also developed a new linear molecular representation for use with LSTMs. When processed with an attention-based bidirectional LSTM, this linear notation proved to be suitable for detecting structural alerts, i.e., parts of the molecules that are related to the biological activity. Originally proposed by Bahdanau et al. (2014), attention-based modeling has gained considerable popularity in the field of deep learning (Luong et al., 2015). When implemented in LSTMs, attention mechanism selectively focuses on certain parts of the input sequence instead of giving equal importance to the whole sequence.

## Materials and Methods

### Data

Three large datasets with significant number of actives were chosen to cover both toxic and pharmacological effects of compounds. The activity outcomes are binary, i.e., active and inactive.

#### Ames Mutagenicity

A database of 23,442 compounds with known Ames test outcome was assembled from various public and proprietary sources. Public sources include Chemical Carcinogenesis Research Information System (CCRIS)^1^, National Toxicology Program (NTP) study data (Tennant, 1991; National Toxicology program), GENE-TOX TOXNET database^2^, Registry of Toxic Effects of Chemical Substances (RTECS^3^; Sweet et al., 1999) and European Food Safety Agency (EFSA)^4^ database. A set of ~12,000 proprietary chemicals provided by the Division of Genetics and Mutagenesis, National Institute of Health Sciences, Japan as part of their Ames/QSAR International Challenge Project (DGM/NIHS, Honma et al., 2019) were also included. Ames test results of these compounds include reverse mutation assay on five sets of bacterial strains recommended by OECD guideline 471 (OECD guideline for testing of chemicals, 1997), with and without S9 metabolic activation, i.e., *S. typhimurium* TA1535, TA1537 or TA97 or TA97a, TA98, TA100, and *E. coli* WP2 uvrA, or *E. coli* WP2 uvrA (pKM101), or *S. typhimurium* TA102 or *E. coli* WP2 or WP2/PKMN1. A compound was categorized as overall Ames negative if it was tested negative against all five strain sets, but positive if it was tested positive in any one of the sets. For Japan NIHS compounds, only class A (strong positives) and class C (clear negatives) were included, class B (weak positives) chemicals were excluded. Upon completion of all the preprocessing steps (described below), the dataset contained 7,260 mutagenic and 11,687 non-mutagenic (total 18,947) chemicals.

#### Inhibitors of Hepatitis C Virus (HCV)

This dataset is from PubChem confirmatory bioassay AID 651820^5^. The aim of this bioassay was to identify novel HCV inhibitors, using a highly sensitive and specific high throughput assay platform which is based on an HCV infectious cell culture system. The original dataset contains 343,600 compounds: 11,664 active, 271,341 inactive, and 60,595 substances with unspecified activity. Only a part of the inactive chemicals was selected randomly to prevent over-representation. After preprocessing, 35,466 chemicals remain (9,935 active and 25,531 inactive).

#### Inhibition of *P. falciparum* Dd2

This dataset is from PubChem primary screening bioassay AID 2302^6^. This assay determines inhibition of *P. falciparum* Dd2 growth by measuring levels of *P. falciparum* lactate dehydrogenase as surrogate of parasite growth. The original dataset contains 13,533 compounds: 7,957 active, 5,489 inactive, and 87 with unspecified activity. After preprocessing, 11,917 chemicals remain. In order to prevent over-representation of active compounds, 4,916 active compounds were randomly selected, and the rest 4,916 inactive compounds were added to them.

#### Data Pre-processing

The datasets used in this study were subjected to some common data preprocessing steps, i.e., aromaticity perception, stereochemistry removal, neutralizing charges on certain atoms and removal of alkali metal salt parts. Only the biggest component of a mixture was retained; in case of duplicates, only one chemical with the highest activity was retained. Chemicals with more than 100-character SMILES code were removed for ease of processing by the LSTM networks since the training data is padded to same length before training, therefore, a few training examples with long SMILES can negatively impact training times. Also, excluding a few big molecules is not an issue when the datasets are already quite large.

The mutagenicity dataset was subjected to some special curation steps. Heavy metals and other known toxic metal salt parts (Pt, Hg, Cd etc.) were retained and joined with their organic counterparts. A mixture or an organic salt was removed if all of its components were present as single compound entries in the data. Remaining mixtures were examined manually and retained only if the mixture's activity was determined to be from a single component. In case of duplicates, only one compound from the set was retained after combining their mutagenicity outcomes.

In order to build and test the QSAR models, the datasets were divided into training and external test sets as shown in Table 1. The test sets were created by randomly taking out 10–20% compounds from the dataset.

**Table 1:** Composition of the training and external test sets.

	**Training data**			**External test data**		
**Data set**	**Active**	**Inactive**	**Total**	**Active**	**Inactive**	**Total**
Ames mutagenicity	6,527	10,478	17,005	732	1,210	1,942
Inhibition of Hepatitis C Virus (HCV)	8,971	22,948	31,919	964	2,583	3,547
Inhibition of *P. falciparum* Dd2	3,948	3,918	7,866	968	998	1,966

### Computer Hardware

Microsoft Windows 10 64-bit OS based desktop computer, 64 GB RAM, 18-core i9-7980XE 2.60 GHz CPU with one NVIDIA GeForce GTX 1080 Ti GPU was used.

### Software

Python version 3.6.6 (Python Software Foundation), Keras (Chollet, 2015), Google TensorFlow (Abadi et al., 2015) and R version 3.5.1 (R Core Team, 2014) was used for implementing various machine learning algorithms including LSTMs. An in-house software library was used for various cheminformatics operations, e.g., handling of chemical structures, molecular fragmentations, and building the fragment-based models.

### Linear Representation of Chemicals

Two types of sequential representation of molecular structures were used for chemical structure input to LSTM neural networks:

#### Simplified Molecular-Input Line-Entry System (SMILES)

We have primarily used canonical SMILES codes in this study (Weininger et al., 1989). However, despite being immensely useful and popular, conventional SMILES contain characters, e.g., ring opening and closing numbers and parenthesis for branches, that are difficult to map back to specific atoms in the chemical structure, a step needed for detecting structural alerts. Also, an atom is represented simply by its elemental symbol in SMILES and the LSTMs will have to keep track of other atoms to learn its characteristics, e.g., hybridization, number of attached hydrogens, ring membership etc. Although LSTMs are very good in recognizing such relationships in the input sequences, there is no particular advantage in making them work harder to learn such basic information in the molecular structures.

#### Molecular Linear Notation by Circular Traverse (MLNCT)

MLNCT is a new linear representation developed to solve the alert detection issues of conventional SMILES. The MLNCT algorithm starts from any heavy atom in the molecular structure and successively travels outwards by one bond in each iteration and records the connected atoms in each step. In the resulting notation, every such step is separated by a space, and atoms are separated by an asterisk within a particular step. Atoms are coded as strings with multiple characters comprised of atom symbol, hybridization, number of hydrogens, number of double or triple bonds, charge, ring membership etc. Every component in a MLNCT code corresponds to one atom in the source molecule and can be traced back if needed. Examples of MLNCT codes of a few substances are shown in Table 2. Recently, O'Boyle and Dalke (2018) developed a new type of SMILES notation which is reportedly more suitable for deep learning of chemical structures, however, this representation was mainly developed to address the problems of invalid SMILES generated by deep neural networks for *de novo* design of molecules and contain parenthesis and ring numbers that are problematic for our purposes.

**Table 2:** Examples of molecular linear notation by circular traverse (MLNCT) coding.

**Compound**	**Canonical SMILES**	MLNCT representation^*^
Alanine	CC(N)C(O) = O	C3H3 C3H N3H2^*^C21 OH^*^O1
Tyrosine	NC(Cc1ccc(O)cc1)C(O) = O	N3H2 C3H C3H2^*^C21 c^*^OH^*^O1 cH^*^cH cH^*^cH c OH
Valsartan	[H][n]1nnc(n1)c2ccccc2c3ccc(CN(C(C(C)C)C(O) = O)C(= O)CCCC)cc3	nH n^*^n n^*^c c cH^*^c cH^*^cH^*^c cH^*^cH^*^cH cH^*^cH c C3H2 N3 C3H^*^C21 C3H^*^C21^*^C3H2^*^O1 C3H3^*^C3H3^*^OH^*^O1^*^C3H2 C3H2 C3H3

* *N3–sp^3^ nitrogen; C3 - sp^3^ carbon; C2–sp^2^ carbon; C1–sp carbon; [c.]–aromatic carbon at a ring joint; [C3^∧^]–sp^3^ carbon in a three- or four-membered ring; C21–sp^2^ carbon with one double bond; O1–Oxygen with one double bond; n–aromatic nitrogen; H2–two hydrogens*.

### Model Building

Four types of models were built for every data set:

1. Long short-term memory (LSTM) models with canonical SMILES (*LSTM_SMILES*).
2. LSTM models with MLNCT codes (*LSTM_MLNCT*).
3. Simple fully connected neural network models with molecular fragment descriptors (*FRAG_NN*).
4. Logistic regression models with molecular fragment descriptors (*FRAG_LOGIST*).

#### Model Building Using LSTMs

**Figure 1:**
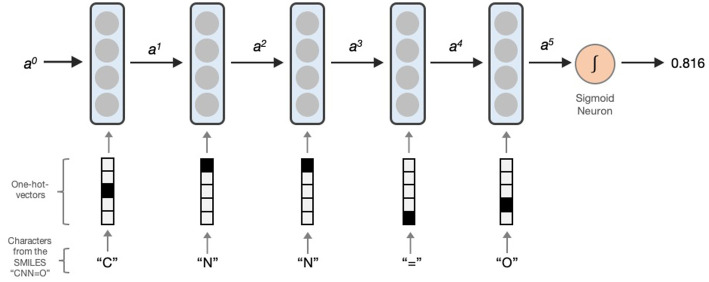
Processing a SMILES code using a unidirectional LSTM network. Activations from the LSTM unit at a particular step is denoted by *a*^*x*^*. a*^0^ is the initial activation and is an array of zeroes. The output is the predicted probability which can be converted to an active/inactive format using a decision threshold.

LSTM networks essentially contain a computing cell which performs a loop of computation with as many steps as the length of the input SMILES or MLNCT for mapping the molecule to the output activity. As illustrated in Figure 1, the cell takes one character of SMILES or an atom string of MLNCT code at every step, and passes the computed activation value to the next and therefore allows information to persist as the whole input sequence is processed, i.e., in producing the output at a certain step, it can use the information from inputs at earlier steps. After the completion of all the steps, the LSTM cell produces a probability value as the output, ranging between 0 and 1. This value can be converted to an active/inactive class using a decision threshold.

##### Pre-processing of LSTM input *sequences*

The LSTM network does not accept the molecular linear representations as it is, rather it takes a sequence of fixed-sized vectors generated from SMILES or MLNCT. Each character in SMILES or an atom string in MLNCT is converted to a “one-hot-vector” in which only one element is 1 and rest are all 0 s, as shown in Figure 1. The length of each one-hot vector is equal to the number of unique components in the training sequences and also referred to as the vocabulary size. Following are relevant details for the two types of sequences used in this study:

1. *SMILES:* Each character of the SMILES code is converted to a one-hot vector. Vocabulary size is 53, 34, and 30 for Ames, Hepatitis C Virus and *P. falciparum* datasets, respectively.
2. *MLNCT:* Each atom string and spaces are converted to one-hot vectors. Any atom type that appears <4 times in the training set is converted to a special type called <*unk*>. The vocabulary size is 106, 72, and 53 for Ames, Hepatitis C Virus and *P. falciparum* datasets, respectively.

During prediction, linear representations of the query chemicals are processed in the same manner. If any previously unseen atom type is encountered in the MLNCT, it is converted to <*unk*>. In case of a SMILES, the query molecule is labeled as out-of-domain if an unseen character is encountered.

##### Training LSTM models

A validation split of 0.1 was used during training, leading to the hold-out of 10% the training examples (i.e., the validation set) that were used for assessment of the model while it is being trained. The validation set prediction results were utilized in tuning the hyperparameters, whereas the test set is used only once after the model is built.

Tuning the values of various hyperparameters is essential for successful training of LSTM networks and to determine the structure of the network. The main hyperparameters are learning rate, number of LSTM hidden neurons, batch size, number of training epochs, and dropout rates. A combination of systematic search and experimentation was used to determine the appropriate value of the hyperparameters. Learning rate was varied from 0.05 to 0.0001 in small intervals (e.g., 0.05, 0.01, 0.005 etc.), number of hidden neurons was varied as 64, 128, 256, 512 etc. and the batch size was varied as 28, 64, 128, 256, 512, and 1,024. Number of training epochs was determined by observing when the prediction accuracy did not improve anymore during training. Dropout rate, which is part of a regularization technique for preventing overfitting, was determined by gradually increasing its value from zero (i.e., 0, 0.1, 0.2, 0.3 etc.) and stopping as soon as the prediction accuracy of the training set and the validation set becomes roughly the same. If the validation accuracy is lower than that of the training set, it usually is a sign of overfitting.

#### Model Building Using Molecular Fragment Descriptors

Fragment descriptor-based models were built as representatives of the conventional descriptor based QSARs. The model building process consists of the following steps:

1. Convert the SMILES of the training chemicals to molecular connectivity tables.
2. Generate extended-connectivity fragment fingerprint (ECFP) style (Rogers and Hahn, 2010) atom centered fragments (from 1 to 5 bonds) from every atom of each training set chemical.
3. Create a fragment count matrix (*X*) for the unique fragments discovered in step 2. The rows of this matrix correspond to the training compounds and the columns correspond to the fragments. The elements of this matrix are the counts of individual fragments in training compounds. *X* is essentially a sparse matrix in which majority of the elements are zeroes. Also, create a column matrix *Y*, containing the activity labels of the training chemicals.
4. Eliminate *X* matrix columns that occur in <5 training compounds to prevent selection of fragments that may cause overfitting. Also, perform descriptor selection to eliminate *X*-matrix columns that are not relevant to the activity in question. We have used the L1 regularization/Lasso regression (Friedman et al., 2008) for this purpose. This usually results in elimination of majority of the columns of the *X* matrix and the resulting matrix is called *X_small*.
5. The final models were built by fitting *Y* and *X_small* using either logistic regression (*FRAG_LOGIST*) or a simple fully connected neural network (*FRAG_NN*). If logistic regression is used, the magnitude of the coefficients of this model indicates the relative importance of the fragment toward the activity. Positive coefficients are referred to as *Alerts* and negative coefficients are called as *Deactivating Features*.

### Alert Identification Using Attention-Based LSTMs

**Figure 2:**
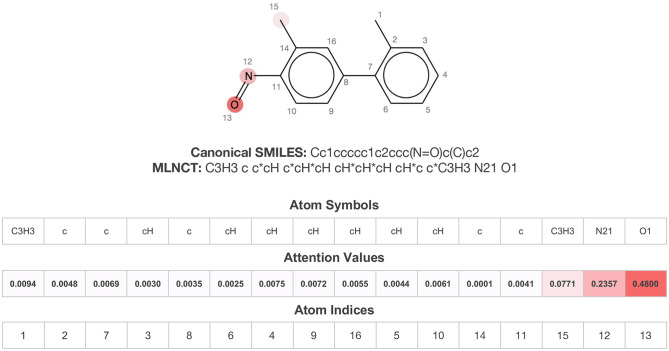
Identification of the mutagenicity structural alert for a query compound using the attention values on the MLNCT code processed through an attention-based bidirectional LSTM model.

As mentioned before, bidirectional attention-based LSTM networks were used for alert detection. It searches through the input SMILES or MLNCT sequence to compute importance of various parts of the sequence toward the activity. A small neural network with one hidden-layer is placed between the encoder and the decoder to accomplish the attention task. If an MLNCT is used as input, the obtained attention values for every component of the MLNCT string was recorded and mapped back into the individual atoms of the query chemical. This process is shown in Figure 2 for an example query chemical, where atoms with the highest attention values accurately correspond to the nitroso group, a known mutagenic functionality.

### Performance Metrics

In order to assess and compare the performance of the models, mainly the area under the curve (*AUC*) from the receiver operating characteristic (*ROC*) curves were used. We also used some standard metrics:

Sensitivity (*SENS*) = *TP/(TP* + *FN)*
Specificity (*SPEC*) = *TN/(TN* + *FP)*
Accuracy (*ACC*) = *(TP* + *TN)/(TP* + *TN* + *FP* + *FN)*
Balanced accuracy (*BAL_ACC*) = *(Sensitivity* + *Specificity)/2*
Positive prediction value (*PPV*) = *TP/(TP* + *FP)*
Negative prediction value (*NPV*) = *TN/(TN* + *FN)*
Coverage (*COVG*) = *Number of query compounds within applicability domain/total number of query compounds*.

Here, *TP, FP, TN, and FN* are the counts of true positive, false positive, true negative and false negative compounds respectively. The *COVG* is only needed when applicability domain conditions are implemented.

The values of various metrics were recorded at specific decision thresholds which were determined for every model from the results of the 10-fold cross validations exercise and the thresholds corresponding to the best *BAL_ACC* were used.

### Model Validation

Following validation protocols were used for evaluating the models:

1. Leave 10% out 10-times cross validation: Ten percent chemicals were removed from the training set; the model was rebuilt with the rest 90% and the excluded chemicals were used as a test set. The process was repeated 10 times.
2. Y-Randomization: In this exercise, the activity labels of the training set were shuffled, model was rebuilt, and the external test set was predicted. This test is performed to determine the models' susceptibility for chance correlations.
3. External test: The compounds that were kept out and never used as part of any training were predicted to check the external predictive ability of the model.
4. Model performance across training set chemical space: In this exercise, the models' ability to predict test compounds with varying degrees of similarity with the training set compounds was evaluated. This test was done for the external sets and for the hold-out sets of the cross-validation exercise. Following are the required steps:
  - Predict the test set using the model in question and record the computed probability for every test compound.
  - Pairwise similarity (using 2 to 8-atom linear fragments hashed to 1,024-bit fingerprints or 166-bit MACCS keys via Tanimoto coefficient) was calculated between each test set compound and all the training compounds.
  - Five most similar training compounds were identified, and their similarity were averaged and assigned to the test chemical as its similarity to the training set.
  - The test set compounds were sorted in ascending order of their similarity to the training set.
  - The test set is scanned starting from the compound with lowest similarity. At every *n*^*th*^ (*n* = 20, 50, 100) compound, its similarity is recorded, and balanced accuracy is computed for the set of compounds scanned so far. This gives the performance of the model for chemicals that have equal or less similarity to the training set.

### Domain of Applicability

We used a combination of two criteria to determine if a query chemical's prediction falls outside the domain of applicability of a model: 1. if the calculated probability is ±0.05 of the decision threshold of the model and, 2. if the query chemical has a functionality that is not present in the training set chemicals. The first condition excludes predictions for which the model has weak differentiability and the second one excludes query chemicals that have structural features for which the model was not trained. The second condition was implemented by creating a dictionary of 3-atom fragments from the training chemicals, and the query chemical is checked during prediction to see if it contains any fragment that is not present in the dictionary. It is classified as out-of-domain, if it contains 3 or more of such “unknown” fragments.

## Results and Discussion

We found that hyperparameter tuning is the most time-consuming part of the LSTM training. Systematic search and some trial and error was needed to find the value of individual hyperparameters that works in combination with others. Also, we found that the required number of epochs is independent of the size of the training set, for example, the *P. falciparum* dataset with 7,866 training compounds needed 10,000 epochs, whereas the Ames dataset with 17,005 training compounds needed only 100 epochs when trained with SMILES codes. Moreover, LSTM models required substantially fewer epochs in training with MLNCT codes as compared to training with SMILES. For example, the Hepatitis C model required 9,000 epochs when trained with SMILES, whereas only 300 epochs were needed for training with MLNCT codes. Therefore, it is reasonable to assume that it is easier for the LSTMs to learn from MLNCT codes than SMILES, possibly due to detailed atom typing. The final hyperparameter values are shown in Tables 3, 4.

**Table 3:** Hyperparameter values for LSTM training using canonical SMILES as input.

**Data set**	**LSTM neurons**	**Dropout rate**	**Number of training epochs**	**Size of validation set**	**Learning rate**	**Batch size**
Ames mutagenicity	256	0.3	100	0.1	0.005	128
Inhibition of Hepatitis C Virus (HCV)	256	0.6	9,000	0.1	0.0001	256
Inhibition of *P. falciparum* Dd2	256	0.6	10,000	0.1	0.0001	256

**Table 4:** Hyperparameter values for LSTM training using MLNCT codes as input.

**Data set**	**LSTM neurons**	**Dropout rate**	**Number of training epochs**	**Size of validation set**	**Learning rate**	**Batch size**
Ames Mutagenicity	256	0.3	170	0.1	0.001	256
Inhibition of Hepatitis C Virus (HCV)	128	0.2	300	0.1	0.0005	128
Inhibition of *P. falciparum* Dd2	512	0.7	2,700	0.1	0.0005	256

For the fragment-based models, variable selection is the most time-consuming step of model building. Some hyperparameters needed to be tuned for the fragment-based neural networks, however, no such optimization was required for the logistic regression models. Training of the neural networks using the fragment descriptors were computationally inexpensive and fast enough to allow rapid hyperparameter tuning. We found a simple network with two hidden layers that works well for all three datasets, with 15 and 7 sigmoid activation neurons and dropout rates of 0.5 and 0.3 for the first and second hidden layer, respectively. The output layer is composed of a single sigmoid neuron. Validation split was set at 0.1; learning rate of 0.001 and 30 epochs were used for the training.

The results of various validation experiments are discussed below. It should be noted that the LSTM models' and fragment-based neural networks' effective training set sizes are always 10% smaller due to the use of validation split during training, which does not happen with the fragment-based logistic regression models.

### Ames Mutagenicity

Results of the leave 10% out 10-times cross validations for all the four mutagenicity models are given in Table 5. We found the optimal value of the decision threshold to be between 0.38 and 0.4. This happens to be close to the ratio of active/inactive in the dataset, which is 0.38.

**Table 5:** Ames mutagenicity models' 10% out 10-times cross validation prediction metrics.

**Metric**	**LSTM_SMILES**	**LSTM_MLNCT**	**FRAG_NN**	**FRAG_LOGIST**
Threshold	0.38	0.40	0.38	0.38
*SENS*	0.841 ± 0.025	0.830 ± 0.023	0.825 ± 0.020	0.833 ± 0.021
*SPEC*	0.892 ± 0.018	0.887 ± 0.023	0.906 ± 0.013	0.896 ± 0.014
*ACC*	0.873 ± 0.009	0.865 ± 0.008	0.875 ± 0.013	0.872 ± 0.014
*BAL_ACC*	0.867 ± 0.010	0.859 ± 0.007	0.866 ± 0.014	0.865 ± 0.015
*PPV*	0.829 ± 0.028	0.825 ± 0.030	0.845 ± 0.024	0.832 ± 0.023
*NPV*	0.901 ± 0.017	0.892 ± 0.012	0.893 ± 0.014	0.896 ± 0.014
*AUC*	0.935 ± 0.009	0.931 ± 0.005	0.936 ± 0.010	0.934 ± 0.010

**Figure 3:**
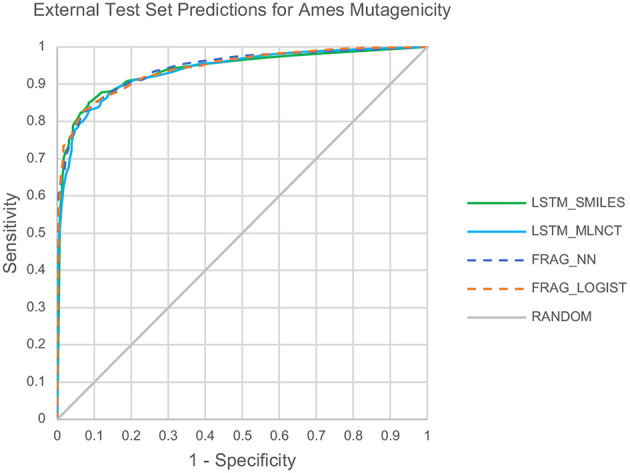
ROC plots for the Ames mutagenicity external test set predictions.

Also, the prediction metrics and the ROC plots for the 1,942 external test chemicals are shown in Table 6 and Figure 3, respectively. All the models gave excellent and almost equal performance. The balanced accuracies of training set predictions are about 3–6% better than that of the test set. Comparing to the current state of the art for predicting Ames mutagenicity, these results can be placed at the very high end of performance scale (Benigni and Bossa, 2019; Honma et al., 2019). *AUC* of the y-randomization exercise is also shown in Table 6, it is evident that the results are close to random for all the models. Details of the y-randomization is provided in the Supplementary Information.

**Table 6:** Ames mutagenicity models' training and test set prediction metrics.

**Metric**	**LSTM_SMILES**		**LSTM_MLNCT**		**FRAG_NN**		**FRAG_LOGIST**	
	**Train**	**Test**	**Train**	**Test**	**Train**	**Test**	**Train**	**Test**
Threshold	0.38	0.38	0.40	0.40	0.38	0.38	0.38	0.38
*SENS*	0.904	0.842	0.878	0.854	0.919	0.829	0.911	0.829
*SPEC*	0.942	0.916	0.904	0.881	0.943	0.920	0.956	0.926
*ACC*	0.927	0.888	0.894	0.871	0.934	0.886	0.939	0.890
*BAL_ACC*	0.923	0.879	0.891	0.867	0.931	0.875	0.934	0.878
*PPV*	0.906	0.859	0.850	0.813	0.909	0.862	0.929	0.872
*NPV*	0.941	0.905	0.922	0.909	0.949	0.899	0.945	0.900
*AUC*	0.972	0.938	0.957	0.936	0.982	0.942	0.986	0.941
*AUC (y-rndmized)*	–	0.501	–	0.501	–	0.533	–	0.500

**Figure 4:**
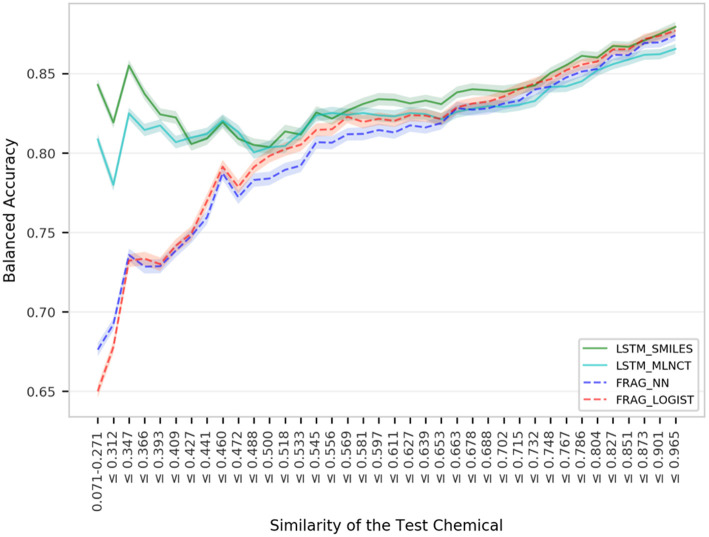
Performance of the mutagenicity models for groups within 1,942 external set compounds with varying similarity with the 17,005 training set chemicals. Each step in the horizontal axis is composed of 50 test compounds. The confidence interval bands around the lines were obtained using a bootstrap resampling process.

Figure 4 shows mutagenicity prediction performance for the external test set across the chemical space defined by the training data. Fragment-based hashed fingerprints were used to compute similarity. Every step in the x-axis consists of 50 test chemicals. It is quite clear that the LSTM models give considerably better results than the fragment-based models for test chemicals with low similarity with the training set. For example, as shown by the very left-end of the plotted lines, test chemicals that have 0.271 or less similarity, *LSTM_SMILES* model is ~15% better than the *FRAG_LOGIST* model. On the other hand, performance of the fragment-based models takes a sharp dip at the left end of the plot. The LSTM models maintain their advantage over the fragment-based models up to a similarity of 0.5. Performance gap between the two types of the models decreases for higher similarity values and the four models show almost equal overall performance (*BAL_ACC* ~ 0.87) as we approach the far right side of the plot. Similar trends were observed when the ten hold-out sets from the 10% out cross validations were subjected to similarity-based performance evaluation. For this, results were averaged from the ten sets and the plots are provided in the Supplementary Information. Improved performance of the LSTMs for low similarity compounds indicate that the LSTMs have better abstraction abilities whereas the fragment descriptor-based models fail to predict compounds that have new features as described by their fragment composition. Analyses using MACCS keys are provided in the Supplementary Information and also show better performance by the LSTM models for compounds with low similarity, but the difference is not as pronounced as that of the fragment-based fingerprints. A possible reason could be due to the diverse nature of the mutagenicity data and presence of many non-drug like compounds (e.g., reagents, impurities etc.), which are not well-represented by the predefined set of MACCS keys. These are important findings because QSAR models often fail in practice when used on novel and structurally different test compounds.

**Figure 5:**
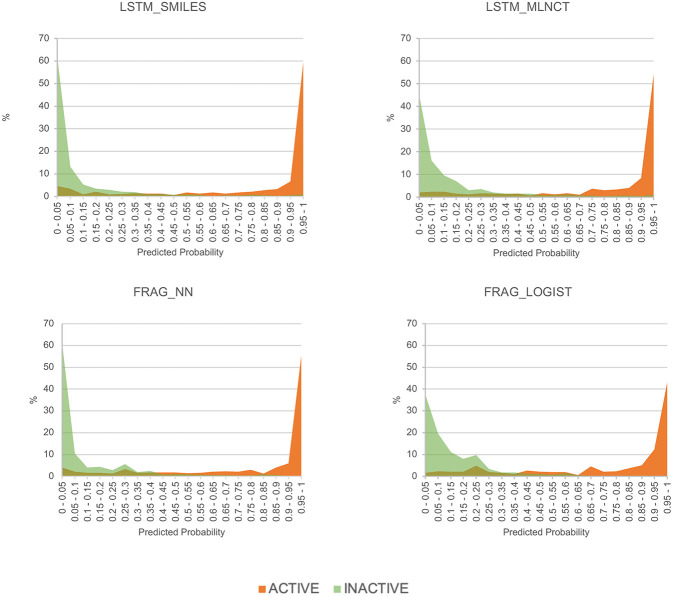
Predicted probability distribution plots for the actives and inactive compounds in the Ames mutagenicity external test set.

To get an alternate view of the mutagenicity models' ability to separate active and inactive compounds, we have plotted the distribution of predicted probabilities of the external set compounds in 20 equally spaced bins between 0 and 1. The plots for the four models are shown in Figure 5. It can be seen that all the models are quite good in separating mutagenic and non-mutagenic chemicals, as active and inactive chemicals are mostly gathered at the right and left side of the plot, respectively, and very few chemicals are present in the middle area.

The prediction metrics and the *ROC* plots give a good idea about the overall model performance for the whole test set; however, they don't show prediction accuracy for different chemical classes present in the test set. For mutagenicity, such chemical classes are fortunately known, mainly due to the work by Benigni (2004), Benigni and Bossa (2006) and others (Plošnik et al., 2016). In this regard, we have divided the external test set of the Ames dataset into 53 different classes and calculated the prediction sensitivity and specificity within each class. Only the results of the classes that have 10 or more test chemicals are shown in Table 7. All the models show good performance across majority of the chemical classes. Such breakdown of performance across different chemical classes is important because low specificity of common toxicity alerts is a known problem (Alves et al., 2016), mainly because they are found in many compounds, both toxic and non-toxic. Primary aromatic amines are one such example with high mutagenicity risk and widely used in chemical synthesis. Mutagenicity prediction of these amines is particularly difficult because the actual reactive species is formed after metabolism (Kuhnke et al., 2019). All our models achieved good sensitivity and specificity for the 135 amines in the external test set with sensitivity between 93 and 95 and specificity between 76 and 85.

**Table 7:** Prediction performance of the mutagenicity models for different chemical classes.

**Chemical class**	**Active/inactive**	**LSTM_SMILES**		**LSTM_MLNCT**		**FRAG_NN**		**FRAG_LOGIST**	
		***SENS***	***SPEC***	***SENS***	***SPEC***	***SENS***	***SPEC***	***SENS***	***SPEC***
Nitro-aromatics	149/30	94	67	98	40	97	70	95	63
Polycyclic aromatics	142/21	98	71	99	67	99	76	99	81
Primary aromatic amines	80/55	93	85	94	76	94	76	94	80
Aliphatic halides	58/36	84	72	79	75	76	69	78	69
Naphthalene analogs	36/43	83	84	89	72	86	86	94	93
Azo-compounds	54/22	87	86	94	77	96	77	94	77
Esters of S(VI)- and P(V)-based acids	26/36	77	86	65	81	77	86	77	86
Three-membered rings, reactive	48/11	88	82	98	64	92	73	96	73
Nitroso compounds	57/0	100	–	98	–	100	–	100	–
Heteroatom-bonded heteroatoms	28/16	100	94	96	69	96	75	96	81
Aromatic acetamides and formamides	27/15	93	87	96	87	96	87	93	80
Aldehydes	13/26	69	85	92	77	85	92	69	96
Haloalkenes	17/21	82	90	82	81	82	76	76	86
Tetrahalogenated aromatics	5/24	40	96	20	96	60	96	40	96
Elementorganics	20/6	90	83	80	67	50	100	50	100
Aromatic N-methyl amines	20/1	90	100	90	100	85	0	85	100
Acyl halides	3/17	67	59	67	65	67	71	67	71
Quinones	14/6	93	67	93	67	100	83	100	83
Poly-carboxyls, phosphates, sulfones	1/18	100	100	100	100	100	89	100	94
Beta-lactams	2/14	100	93	50	93	100	93	100	100
Nitrogen and sulfur mustards	15/0	100	–	100	–	100	–	100	–
Carbamates	7/6	57	83	71	100	86	83	86	83
Purine nucleotide analogs	4/9	75	89	50	89	75	67	75	67
alpha, beta-Unsaturated lactones	7/5	86	80	86	100	100	100	100	100
Fluorenes	9/1	100	100	100	100	100	100	100	100

### Inhibitors of Hepatitis C Virus (HCV)

**Figure 6:**
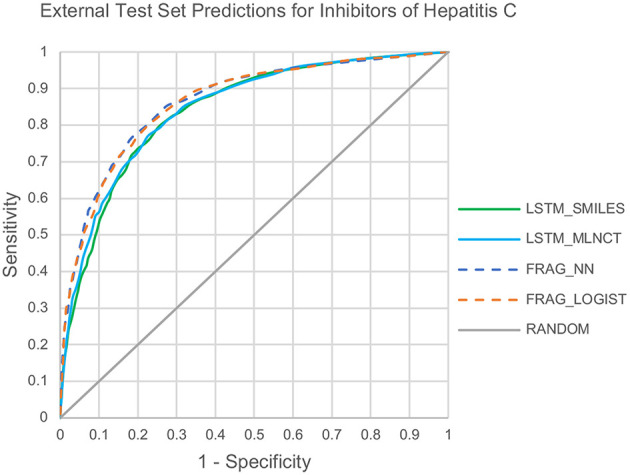
ROC plots for the Hepatitis C Virus external test set predictions.

The cross-validation results are given in Table 8. The decision threshold ranges from 0.22 to 0.28 for different models, and again close to the ratio of active/inactive in the dataset (0.28). The prediction metrics and the ROC plots for the 3,547 external test chemicals are shown in Table 9 and Figure 6, respectively. QSAR models for this dataset have been reported by other researchers (Zakharov et al., 2014), who used a large number of descriptors to build the model, and the highest balanced accuracy reported by them is 0.78 which is very similar to what we are reporting in this work. Like the other two datasets, the training set accuracies are about 4–10% more than the test set accuracies, the MLNCT-based model showed smallest gap, whereas the fragment-based model has the highest gap which may be an indication of overfitting. Similar to the mutagenicity dataset, *AUCs* from the y-randomizations is shown in Table 9, and the results are close to random. Details of y-randomizations and plots of the external test set's probability distribution of predictions are given in the Supplementary Information.

**Table 8:** Hepatitis C Virus (HCV) models' 10% out 10-times cross validation prediction metrics.

**Metric**	**LSTM_SMILES**	**LSTM_MLNCT**	**FRAG_NN**	**FRAG_LOGIST**
Threshold	0.22	0.24	0.22	0.28
*SENS*	0.797 ± 0.028	0.746 ± 0.051	0.760 ± 0.014	0.760 ± 0.017
*SPEC*	0.752 ± 0.033	0.767 ± 0.041	0.811 ± 0.011	0.815 ± 0.007
*ACC*	0.765 ± 0.018	0.761 ± 0.016	0.796 ± 0.006	0.799 ± 0.007
*BAL_ACC*	0.774 ± 0.009	0.757 ± 0.008	0.785 ± 0.005	0.788 ± 0.009
*PPV*	0.560 ± 0.025	0.556 ± 0.038	0.615 ± 0.016	0.620 ± 0.014
*NPV*	0.904 ± 0.011	0.887 ± 0.018	0.895 ± 0.007	0.895 ± 0.006
*AUC*	0.851 ± 0.008	0.831 ± 0.007	0.864 ± 0.005	0.865 ± 0.006

**Table 9:** Hepatitis C Virus (HCV) data set's training and external test set prediction metrics.

**Metric**	**LSTM_SMILES**		**LSTM_MLNCT**		**FRAG_NN**		**FRAG_LOGIST**	
	**Train**	**Test**	**Train**	**Test**	**Train**	**Test**	**Train**	**Test**
Decision threshold	0.22	0.22	0.24	0.24	0.22	0.22	0.28	0.28
*SENS*	0.850	0.811	0.841	0.813	0.904	0.758	0.887	0.742
*SPEC*	0.776	0.728	0.773	0.724	0.881	0.821	0.870	0.818
*ACC*	0.797	0.751	0.792	0.749	0.888	0.804	0.875	0.798
*BAL_ACC*	0.813	0.770	0.807	0.769	0.893	0.790	0.879	0.780
*PPV*	0.597	0.527	0.591	0.524	0.749	0.613	0.728	0.604
*NPV*	0.930	0.912	0.925	0.912	0.959	0.901	0.952	0.895
*AUC*	0.892	0.841	0.887	0.845	0.959	0.864	0.950	0.863
*AUC (y-rndmized)*	–	0.500	–	0.500	–	0.455	–	0.483

**Figure 7:**
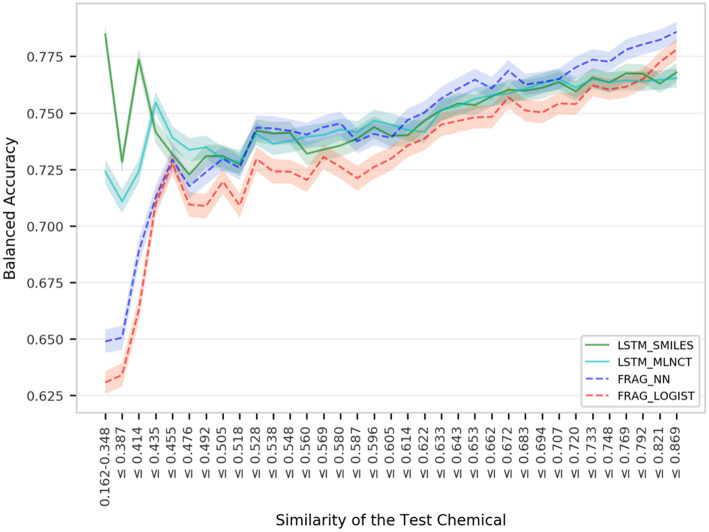
Performance of the Hepatitis C Virus models for igroups within 3,547 external set compounds with different similarity with the 31,919 training set chemicals. Each step in the horizontal axis is composed of 100 test compounds. The confidence interval bands around the lines were obtained using a bootstrap resampling process.

The models are almost equal to each other in terms of overall predictions. However, similar to the mutagenicity dataset, LSTM models give better results than the fragment-based models for test chemicals with low similarity with the training set as shown in the Figure 7. Particularly, the prediction of the *LSTM_SMILES* is ~15% better than the *FRAG_LOGIST* for test chemicals with similarity of 0.348 or less. Results of the 10 hold-out sets from the cross-validation experiments also showed better performance by the LSTM models for low similarity test compounds. Averaged results from the 10 sets are provided in the Supplementary Information. Predefined MACCS keys did not show (provided in the Supplementary Information) significantly better performance by the LSTM models for compounds with low similarity.

### Inhibitors of *P. falciparum* Dd2

**Figure 8:**
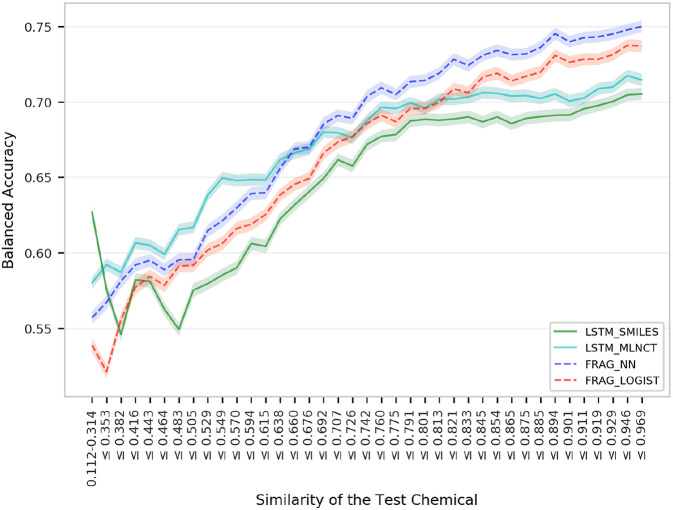
Performance of the *P. falciparum* models for groups within 1,966 external set compounds with different similarity with the 7,866 training set chemicals. Each step in the horizontal axis is composed of 50 test compounds. The confidence interval bands around the lines were obtained using a bootstrap resampling process.

This is the smallest dataset used in this study with 7,866 training compounds. The summary of the cross validation and external set prediction results are shown in Table 10. The decision thresholds range from 0.40 to 0.46. Overall prediction results for the fragment-based models are better than the LSTM models. A possible reason for the lower overall accuracy of the LSTM models could be the relatively smaller size of this dataset, and possibly LSTM networks need large number of examples to learn long-range relationships in the training sequences. The LSTM models perform slightly better with test chemicals that have low similarity with the training set (i.e., similarity of 0.314 or less) as shown in Figure 8. Results from the 10 hold-out sets from the cross-validation experiments also showed slightly better performance by the LSTM models for low similarity test compounds, however. Averaged results from the 10 sets are provided in the Supplementary Information. Detailed results of all the validations for this data set are also provided in the Supplementary Information.

**Table 10:** Summary of the results of various validation exercise performed on the models of the *P. falciparum* dataset.

**Metric**	***LSTM_SMILES***	***LSTM_MLNCT***	***FRAG_NN***	***FRAG_LOGIST***
Threshold	0.46	0.42	0.40	0.46
*Cross-Validation BAL_ACC*	0.715 ± 0.014	0.710 ± 0.015	0.760 ± 0.015	0.759 ± 0.017
*External Set BAL_ACC*	0.707	0.715	0.752	0.741

### Implementation of Model Applicability Domains

We observed about 1–4% increase in the accuracy of the test set predictions across all the models when domain applicability conditions were imposed. At the same time, the coverage was reduced by about 2–13%, i.e., about 98–87% test chemicals were successfully predicted. It is reasonable to assume that high coverage is due to the large sizes of the training sets and good active-inactive separation in the predictions. The data for model performances after employing domain applicability is provided in the Supplementary Information.

### Learning From Non-canonical SMILES

**Figure 9:**
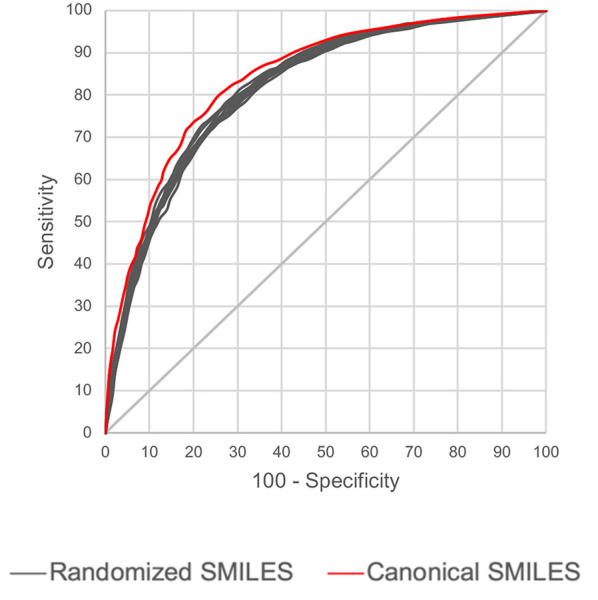
Comparison of prediction performance of LSTM models built using canonical and randomized SMILES. Hepatitis C data test set was used.

We also studied if LSTMs can be trained with SMILES code that are generated from molecular graphs with non-canonical atom ordering and to check if different SMILES representations of a chemical produce wildly different predictions. We have used the Hepatitis C data set to investigate, due to its larger size. A technique was implemented which randomizes the order of atoms in a molecular graph before generating the SMILES code. First, such randomized SMILES were generated for the 31,919 training chemicals, and a unidirectional LSTM network was trained. Thereafter, 10 sets were produced from the external test set which are only different in their SMILES representation but contain exactly the same 3,547 test chemicals. These 10 test sets were predicted, and the ROCs were compared with the ROC from the canonical SMILES, the results are shown in Figure 9. It is apparent that prediction results between the 10 test sets do not fluctuate significantly. Also, the *AUCs* obtained from the non-canonical SMILES are slightly lower than the canonical SMILES, however, the difference is small. One advantage of such robustness is that users of these QSAR models do not need to use SMILES adhering to any particular standard format, potentially increasing usability.

### Comparison of Mutagenicity Alerts Identified by the LSTM and Fragment-Based Modeling

We compared the mutagenicity alerts identified by the attention-based LSTM and the fragment-based models. As discussed earlier, attention values recorded during the prediction were used to detect parts of the input sequence that are important for the computed outcome. A set of 9 compounds with known Ames outcome were used as examples. A comparison is shown in Table 11 between alerts from the *FRAG_LOGIST* and *LSTM_MLNCT* models and Table 12 shows the attention values when SMILES were used as inputs. As mentioned before, MLNCT coding is suitable for alert identification, as every component of an MLNCT string corresponds to an atom in the molecule. In Table 11, any atom that has an attention value of 0.1 or more was considered as part of an alert. The alerts from *FRAG_LOGIST* differentiate between activating and deactivating features as shown in orange and blue color, respectively, whereas, the LSTM_MLNCT alerts only convey if a particular atom is important for the prediction outcome. It is quite clear that the alerts obtained from these two very different types of modeling methods largely agree with each other. The LSTM alerts are not as explicit as the fragment alerts, but they are quite clear in terms of which part of the query compound is key to its toxicity.

**Table 11:** Comparison of mutagenicity alerts identified by the fragment-based and attention-based LSTM mutagenicity models.

**CAS RN#**	**Exptl. Ames outcome**	**Predicted Ames outcome**	**Structural alerts from the fragment-based model**	**Structural alerts from the attention-based LSTM model**
113698-22-9	Positive	Positive	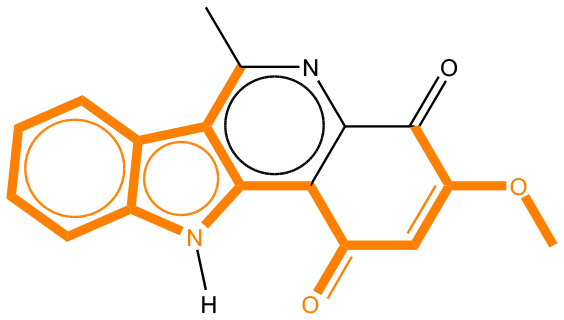	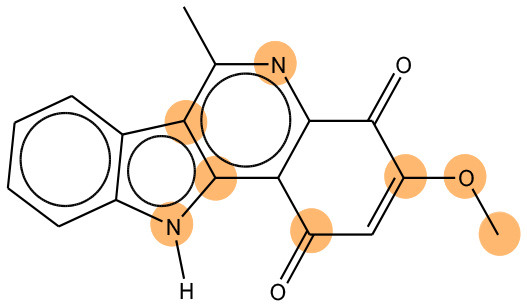
698-63-5	Positive	Positive	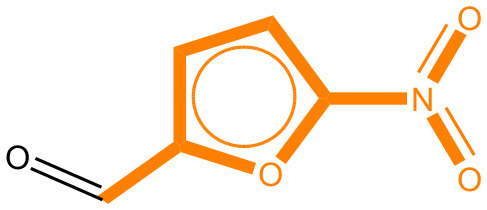	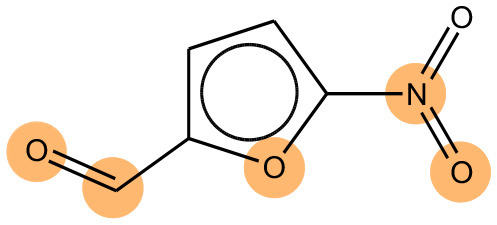
4559-64-2	Positive	Positive	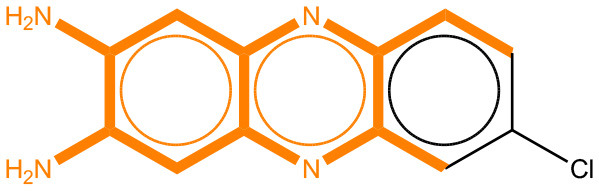	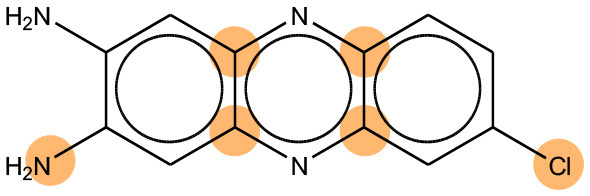
1162-65-8	Positive	Positive	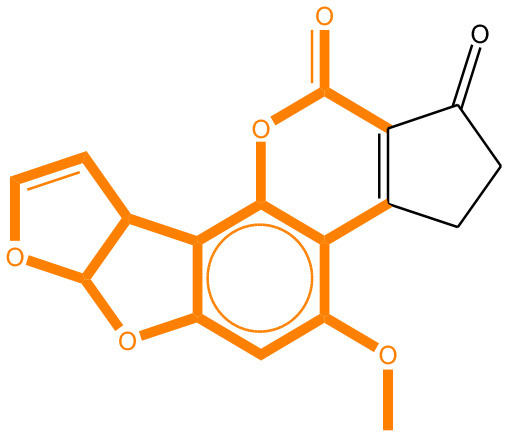	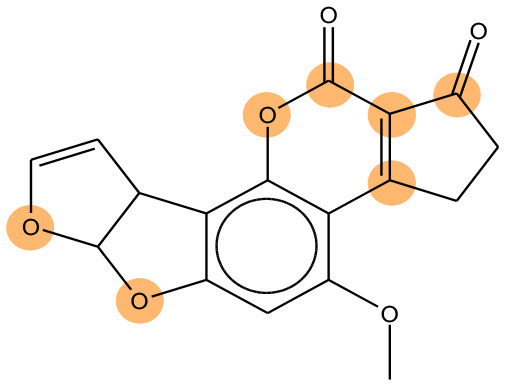
72505-65-8	Positive	Positive	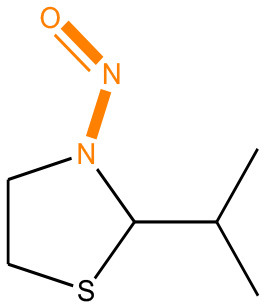	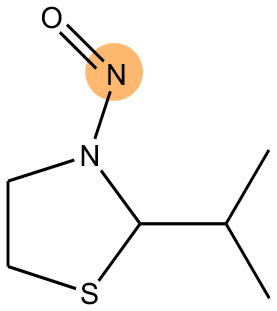
70786-64-0	Positive	Positive	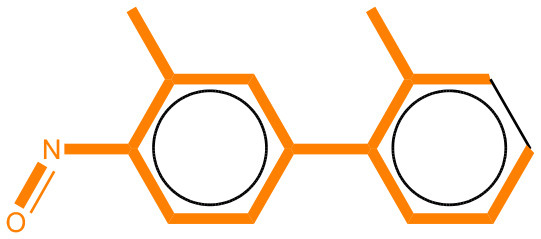	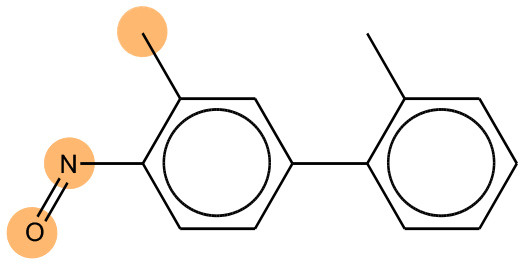
108283-48-3	Positive	Positive	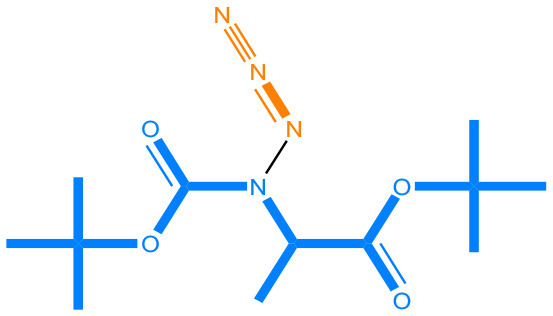	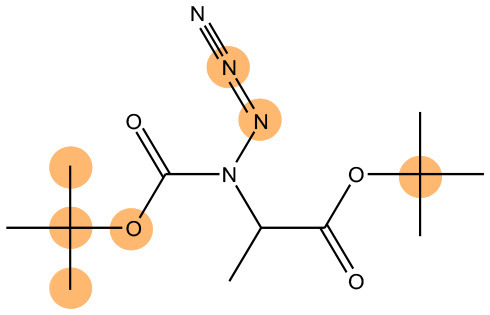
65520-53-8	Positive	Positive	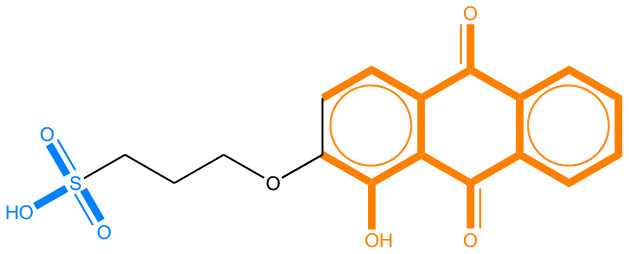	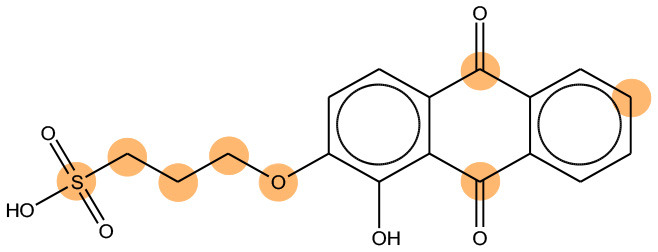
202483-62-3	Negative	Negative	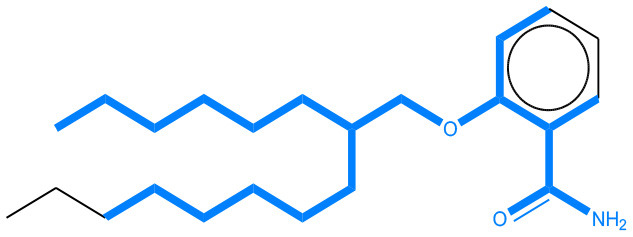	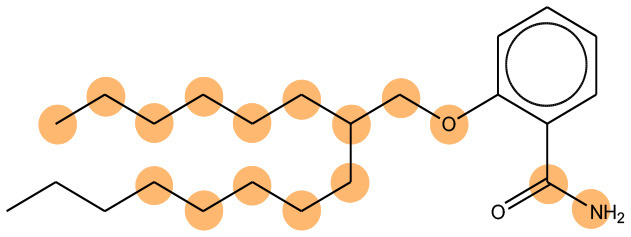

*For the fragment-based model, activating, and deactivating features are shown in orange and blue color, respectively*.

**Table 12:** Attention values for characters of SMILES strings of test chemicals when processed through the attention-based LSTM model.

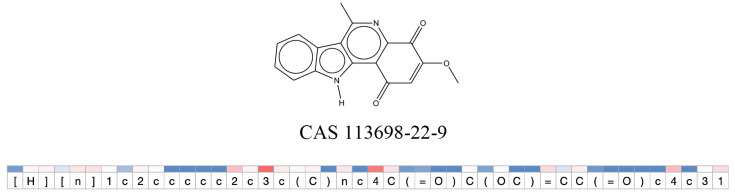
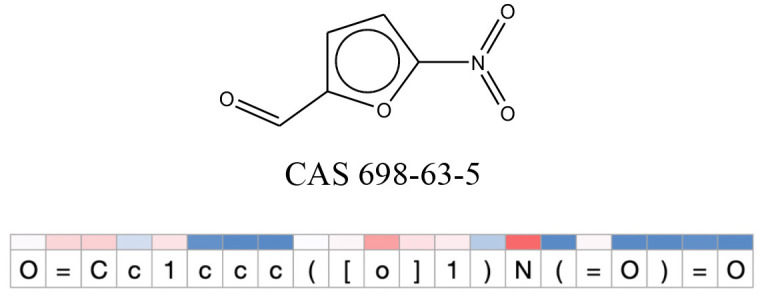
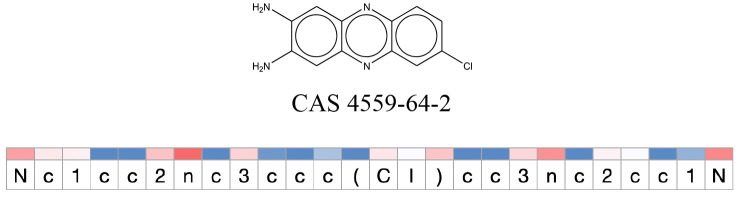
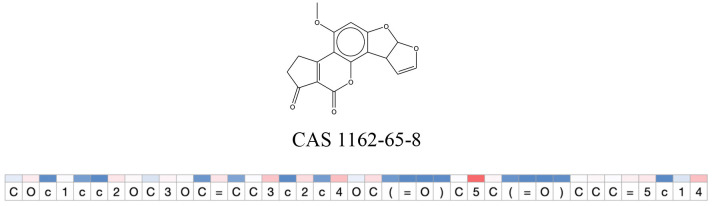
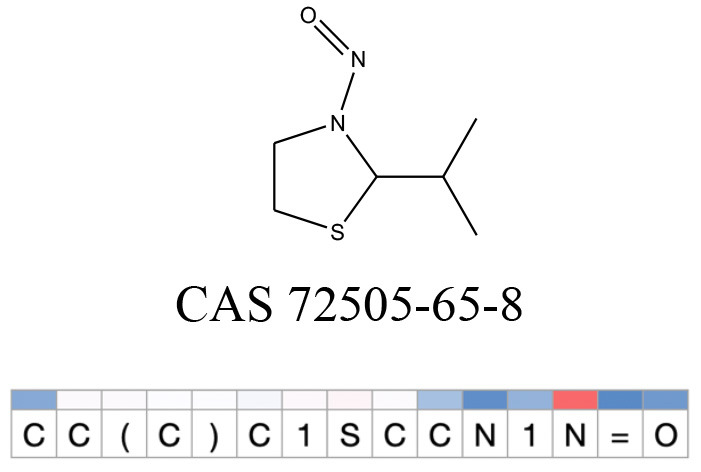
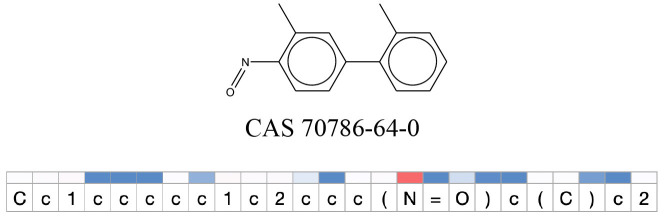
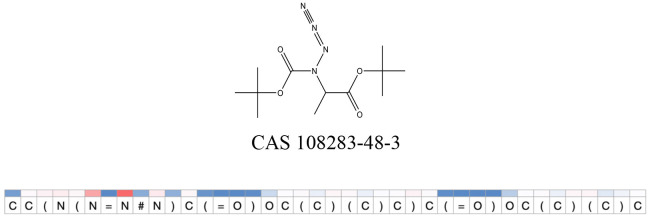
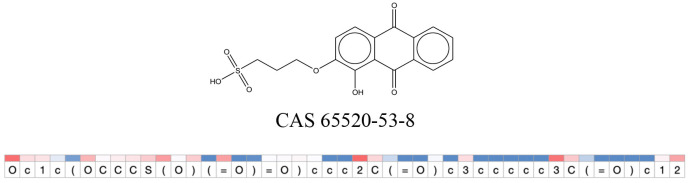
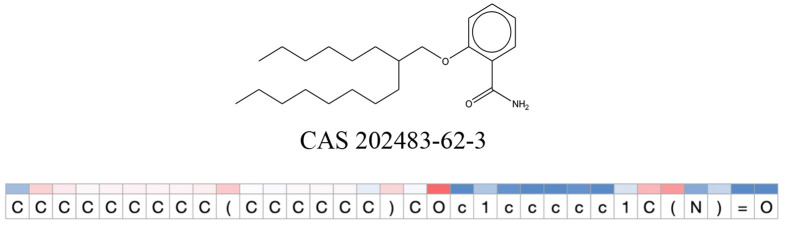

*Red and blue colors indicate high and low attention values respectively*.

After using SMILES as input to the attention-based LSTM, individual characters of the SMILES code were color coded based on their attention-values (Table 12). Red and blue colors indicate high and low attention values, respectively. Such color coding of characters of SMILES code give a good idea about which atoms are important for the prediction outcome but sometimes non-atom characters, i.e., parenthesis or ring closing numbers, also receive high attention values. This makes locating the alerts from the SMILES string difficult in comparison to that of the MLNCT codes.

## Conclusions

We demonstrated a way to build and use QSAR models directly from linear textual representations of chemical compounds, without computing any molecular descriptors. This was achieved via deep learning using LSTM networks. The proposed methodology eliminates some of the difficulties associated with traditional descriptor-based QSAR modeling, e.g., challenges of computing good and relevant descriptors for the endpoint at hand, descriptor selection steps, difficulties associated with interpretation of algorithmically selected descriptors for the target. We have also showed that it is quite possible to detect structural alerts in the query compounds tested by LSTM models, which would be helpful in interpreting results from such descriptor free QSARs.

When compared with a traditional fragment descriptor-based method, the overall performance metrics of the LSTM models showed more or less similar accuracies for three selected end-points, namely Ames mutagenicity, inhibition of *P. falciparum* Dd2 and inhibition of Hepatitis C Virus with training sets of size 17,005, 7,866, 31,919 compounds, respectively. However, LSTM-based models consistently performed better for test chemicals that have low similarity with the training set chemicals.

The results are one step forward toward a time when a list of SMILES codes of chemicals tested in an assay can be used directly to build QSARs using freely available software that has nothing to do with chemistry, potentially expanding the QSAR practitioner base. In addition, descriptor-less QSARs seem to require less domain knowledge, more scalable and can take advantage of the ever-increasing computing power.

### Limitations

Current limitations and weaknesses of the shown methodology include absence of stereochemical information in the input sequences and truncation of salt parts and mixtures, however, it is technically possible to bypass this limitation. Also, we have used only big training sets and therefore, attempts of QSAR modeling with small training sets using these techniques may fail.

### Future Goals

Our future research plans include developing better applicability domain criteria for the descriptor-less models, inclusion of stereo isomers, salts and mixtures in the training sets, building models using small training sets, exploring alternatives for the one-hot representation for the input sequences and investigating necessary steps for regulatory acceptance of such methods.

## Data Availability

The mutagenicity data set used in this study will not be made publicly available because some of the data is proprietary of Nature and can't be made available. Requests to access these datasets should be directed to the corresponding author. The rest of the data analyzed in this study can be found in PubChem AID 651820 and AID 2302.

## Author Contributions

Main concept and experimental design was created by SC. SA implemented the machine learning algorithms and performed the experiments. SC developed and programmed the cheminformatics part and compiled and analyzed the data. Manuscript was primarily written by SC while both authors reviewed and revised the manuscript.

### Conflict of Interest Statement

SC and SA were employed by company MultiCASE Inc.

## Footnotes

^1^CCRIS (Chemical Carcinogenesis Research Information System). Available online at: https://toxnet.nlm.nih.gov/newtoxnet/ccris.htm (accessed April 28, 2019).

^2^GENE-TOX TOXNET Database. Available online at: https://toxnet.nlm.nih.gov/cgi-bin/sis/htmlgen?GENETOX (accessed April 28, 2019).

^3^RTECS (Registry of Toxic Effects of Chemical Substances), Available online at: http://accelrys.com/products/collaborative-science/databases/bioactivity-databases/rtecs.html (accessed April 28, 2019).

^4^EFSA (European Food Safety Agency). *Database Specific for the Pesticide Active Substance and Their Metabolites, Comprising the Main Genotoxicity Endpoints*. Available online at: https://data.europa.eu/euodp/data/dataset/database-pesticide-genotoxicity-endpoints (accessed April 28, 2019).

^5^AID 651820. *PubChem BioAssay Record*. Available online at: https://pubchem.ncbi.nlm.nih.gov/bioassay/651820 (accessed April 28, 2019).

^6^AID 2302. *PubChem BioAssay Record*. Available online at: https://pubchem.ncbi.nlm.nih.gov/bioassay/2302 (accessed April 28, 2019).
