# Supplementary material for: Descriptor Free QSAR Modeling Using Deep Learning With Long Short-Term Memory Neural Networks
Source: Front Artif Intell. 2019 Sep 6;2:17. doi: 10.3389/frai.2019.00017 (PMC7861338; doi:10.3389/frai.2019.00017)
Supplement: Supplementary file 1 [file Data_Sheet_1.docx]

**Descriptor Free QSAR Modeling Using Deep Learning with Long Short-Term Memory Neural Networks**

Suman K. Chakravarti* and Sai Radha Mani Alla

**Supplementary Information**

**Figure S1.** An example of a search to find the optimum value for the learning rate hyperparameter for the LSTM modeling of Ames Mutagenicity using unique SMILES as input.

**Figure S2.** An example of a search to find the optimum value for the number of hidden neurons hyperparameter for the LSTM modeling of Ames Mutagenicity using unique SMILES as input.

**Figure S3.** An example of a search to find the optimum value for the number of training epochs for the LSTM modeling of Ames Mutagenicity using unique SMILES as input.

**Table S1.** Ames mutagenicity data set’s y-randomization prediction metrics by the two LSTM and the two fragment-based models.

| Metric | LSTM_SMILES | LSTM_MLNCT | FRAG_NN | FRAG_LOGIST |
| --- | --- | --- | --- | --- |
| Threshold | 0.38 | 0.40 | 0.38 | 0.38 |
| *SENS* | 0.001 | 0.000 | 0.448 | 0.523 |
| *SPEC* | 0.999 | 1.000 | 0.591 | 0.473 |
| *ACC* | 0.623 | 0.623 | 0.537 | 0.492 |
| *BAL_ACC* | 0.500 | 0.500 | 0.520 | 0.498 |
| *PPV* | 0.500 | - | 0.399 | 0.375 |
| *NPV* | 0.623 | 0.623 | 0.639 | 0.621 |
| *AUC* | 0.501 | 0.501 | 0.533 | 0.500 |

**Table S2.** Hepatitis C Virus (HCV) data set’s y-randomization prediction metrics by the two LSTM and the two fragment-based models.

| Metric | LSTM_SMILES | LSTM_MLNCT | FRAG_NN | FRAG_LOGIST |
| --- | --- | --- | --- | --- |
| Threshold | 0.22 | 0.24 | 0.22 | 0.28 |
| *SENS* | 1.000 | 1.000 | 0.751 | 0.442 |
| *SPEC* | 0.000 | 0.000 | 0.154 | 0.541 |
| *ACC* | 0.272 | 0.272 | 0.316 | 0.514 |
| *BAL_ACC* | 0.500 | 0.500 | 0.452 | 0.491 |
| *PPV* | 0.272 | 0.272 | 0.249 | 0.264 |
| *NPV* | - | 1.000 | 0.623 | 0.722 |
| *AUC* | 0.500 | 0.500 | 0.455 | 0.483 |

| LSTM_SMILES | LSTM_MLNCT |
| --- | --- |
|  |  |
| FRAG_NN | FRAG_LOGIST |
|  |  |
| 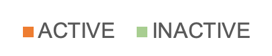 | |

**Figure S4.** Predicted probability distribution plots for the actives and inactive compounds in the Hepatitis C Virus test set using the LSTM and the fragment-based models.

**Table S3.** *P. falciparum* Dd2 data set’s leave 10% out 10-times cross validation prediction metrics by the LSTM and the fragment-based models.

| Metric | LSTM_SMILES | LSTM_MLNCT | FRAG_NN | FRAG_LOGIST |
| --- | --- | --- | --- | --- |
| Threshold | 0.46 | 0.42 | 0.4 | 0.46 |
| *SENS* | 0.755±0.038 | 0.772±0.034 | 0.818±0.021 | 0.780±0.017 |
| *SPEC* | 0.676±0.048 | 0.648±0.032 | 0.701±0.029 | 0.738±0.028 |
| *ACC* | 0.715±0.015 | 0.711±0.016 | 0.761±0.015 | 0.759±0.017 |
| *BAL_ACC* | 0.715±0.014 | 0.710±0.015 | 0.760±0.015 | 0.759±0.017 |
| *PPV* | 0.706±0.029 | 0.690±0.023 | 0.738±0.019 | 0.754±0.025 |
| *NPV* | 0.729±0.032 | 0.739±0.021 | 0.789±0.024 | 0.765±0.022 |
| *AUC* | 0.788±0.014 | 0.786±0.016 | 0.840±0.014 | 0.838±0.016 |

**Table S4.** *P. falciparum* Dd2 data set’s y-randomization prediction metrics by the LSTM and the fragment-based models.

| Metric | LSTM_SMILES | LSTM_MLNCT | FRAG_NN | FRAG_LOGIST |
| --- | --- | --- | --- | --- |
| Threshold | 0.46 | 0.42 | 0.4 | 0.46 |
| *SENS* | 0.852 | 1.000 | 1.000 | 0.616 |
| *SPEC* | 0.134 | 0.000 | 0.000 | 0.366 |
| *ACC* | 0.488 | 0.492 | 0.492 | 0.489 |
| *BAL_ACC* | 0.493 | 0.500 | 0.500 | 0.491 |
| *PPV* | 0.488 | 0.492 | 0.492 | 0.485 |
| *NPV* | 0.484 | - | - | 0.495 |
| *AUC* | 0.508 | 0.500 | 0.553 | 0.491 |

**Table S5.** *P. falciparum* Dd2 data set’s training and test set prediction metrics by the LSTM and the fragment-based models.

| Metric | LSTM_SMILES | | LSTM_MLNCT | | FRAG_NN | | FRAG_LOGIST | |
| --- | --- | --- | --- | --- | --- | --- | --- | --- |
|  | Train | Test | Train | Test | Train | Test | Train | Test |
| Threshold | 0.46 | 0.46 | 0.42 | 0.42 | 0.40 | 0.40 | 0.46 | 0.46 |
| *SENS* | 0.838 | 0.733 | 0.869 | 0.802 | 0.916 | 0.805 | 0.899 | 0.770 |
| *SPEC* | 0.764 | 0.681 | 0.692 | 0.629 | 0.803 | 0.700 | 0.831 | 0.711 |
| *ACC* | 0.801 | 0.707 | 0.781 | 0.714 | 0.860 | 0.752 | 0.865 | 0.741 |
| *BAL_ACC* | 0.801 | 0.707 | 0.780 | 0.715 | 0.859 | 0.752 | 0.865 | 0.741 |
| *PPV* | 0.782 | 0.691 | 0.740 | 0.677 | 0.824 | 0.722 | 0.842 | 0.721 |
| *NPV* | 0.824 | 0.725 | 0.839 | 0.766 | 0.905 | 0.787 | 0.891 | 0.762 |
| *AUC* | 0.885 | 0.782 | 0.872 | 0.790 | 0.945 | 0.831 | 0.944 | 0.829 |
| *AUC (y-rndmized)* | - | 0.508 | - | 0.500 | - | 0.553 | - | 0.491 |

**Figure S5.** ROC plots for the *P. falciparum* Dd2 test set predictions using the LSTM and the fragment-based models.

| LSTM_SMILES | LSTM_MLNCT |
| --- | --- |
|  |  |
| FRAG_NN | FRAG_LOGIST |
|  |  |
| 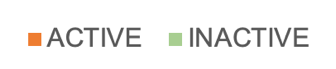 | |

**Figure S6.** Predicted probability distribution plots for the actives and inactive compounds in the *P. falciparum* Dd2 test set using the LSTM and the fragment-based models.

**Figure S7.** Similarity based performance of the mutagenicity models for the 10 hold-out test sets from the leave 10% out cross-validations. Each step in the horizontal axis is composed of 50 test compounds.

**Figure S8.** Similarity based performance of the Hepatitis C Virus models for the 10 hold-out test sets from the leave 10% out cross-validations. Each step in the horizontal axis is composed of 100 test compounds.

**Figure S9.** Similarity based performance of the *P. falciparum* models for the 10 hold-out test sets from the leave 10% out cross-validations. Each step in the horizontal axis is composed of 20 test compounds.

**
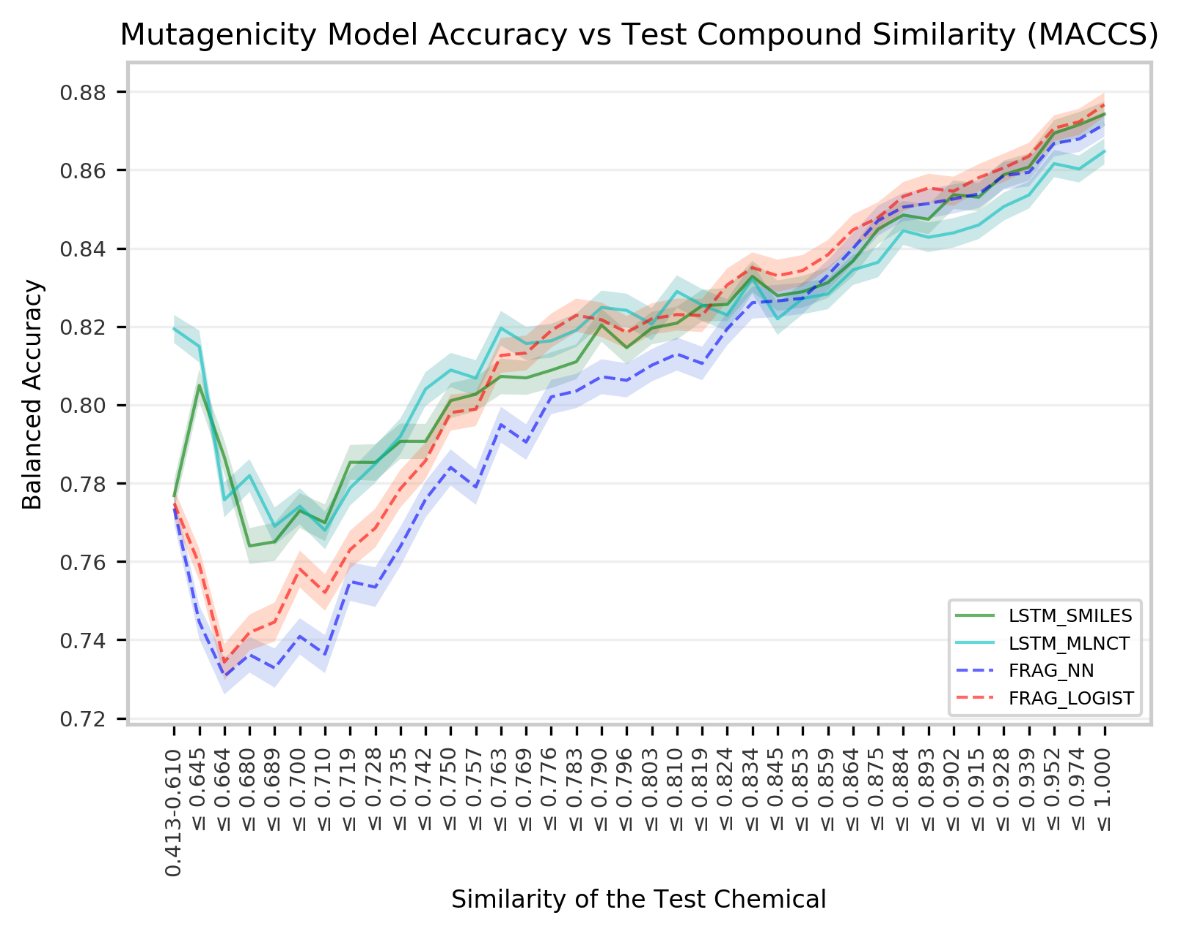
**

**Figure S10.** MACCS key similarity-based performance of the mutagenicity models for 1942 external set compounds the 17005 training set chemicals. Each step in the horizontal axis is composed of 50 test compounds. The confidence interval band around the lines were obtained using a bootstrap sampling process.


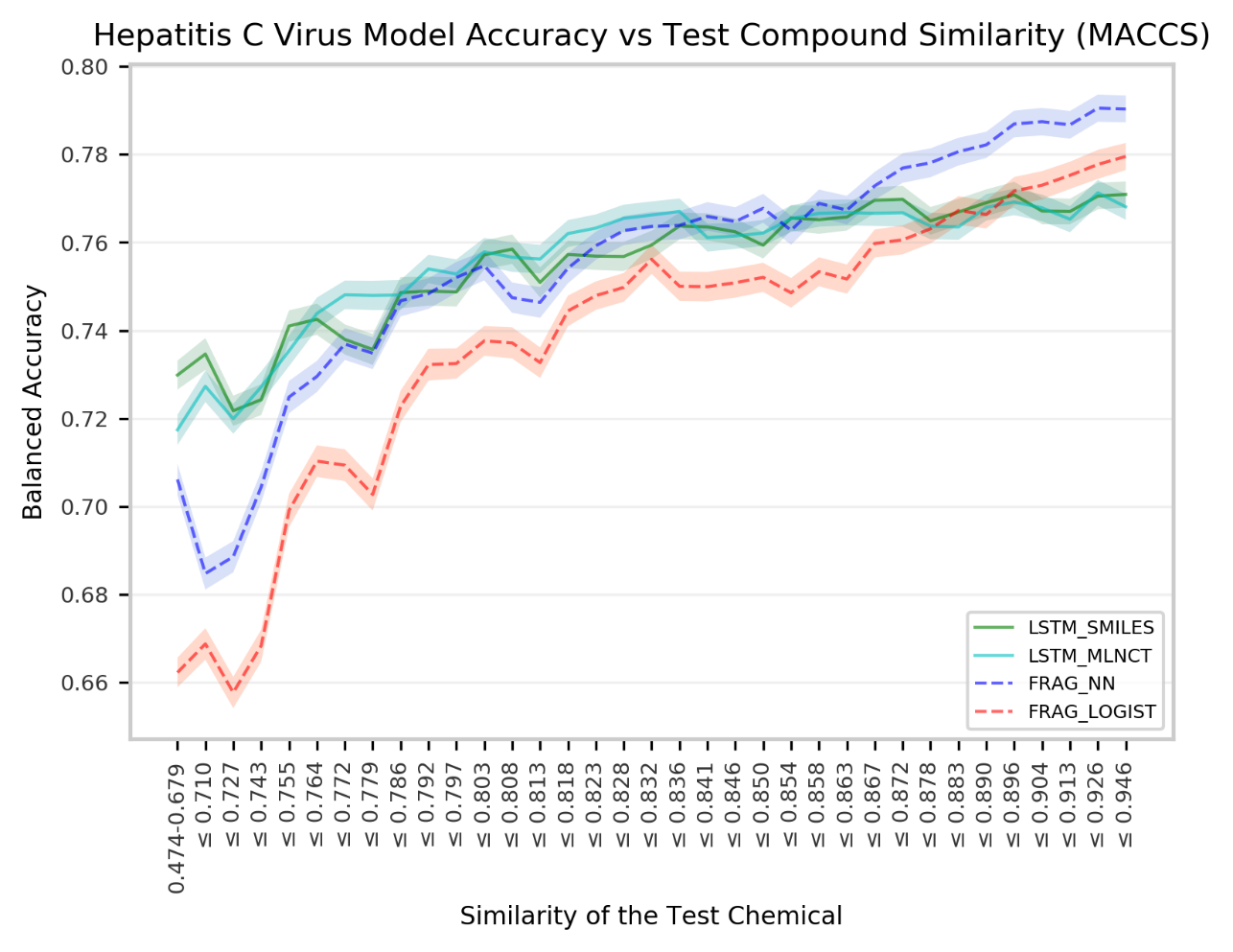


**Figure S11.** MACCS key similarity-based performance of the Hepatitis C virus models for 3547 external set compounds the 31919 training set chemicals. Each step in the horizontal axis is composed of 100 test compounds. The confidence interval band around the lines were obtained using a bootstrap sampling process.


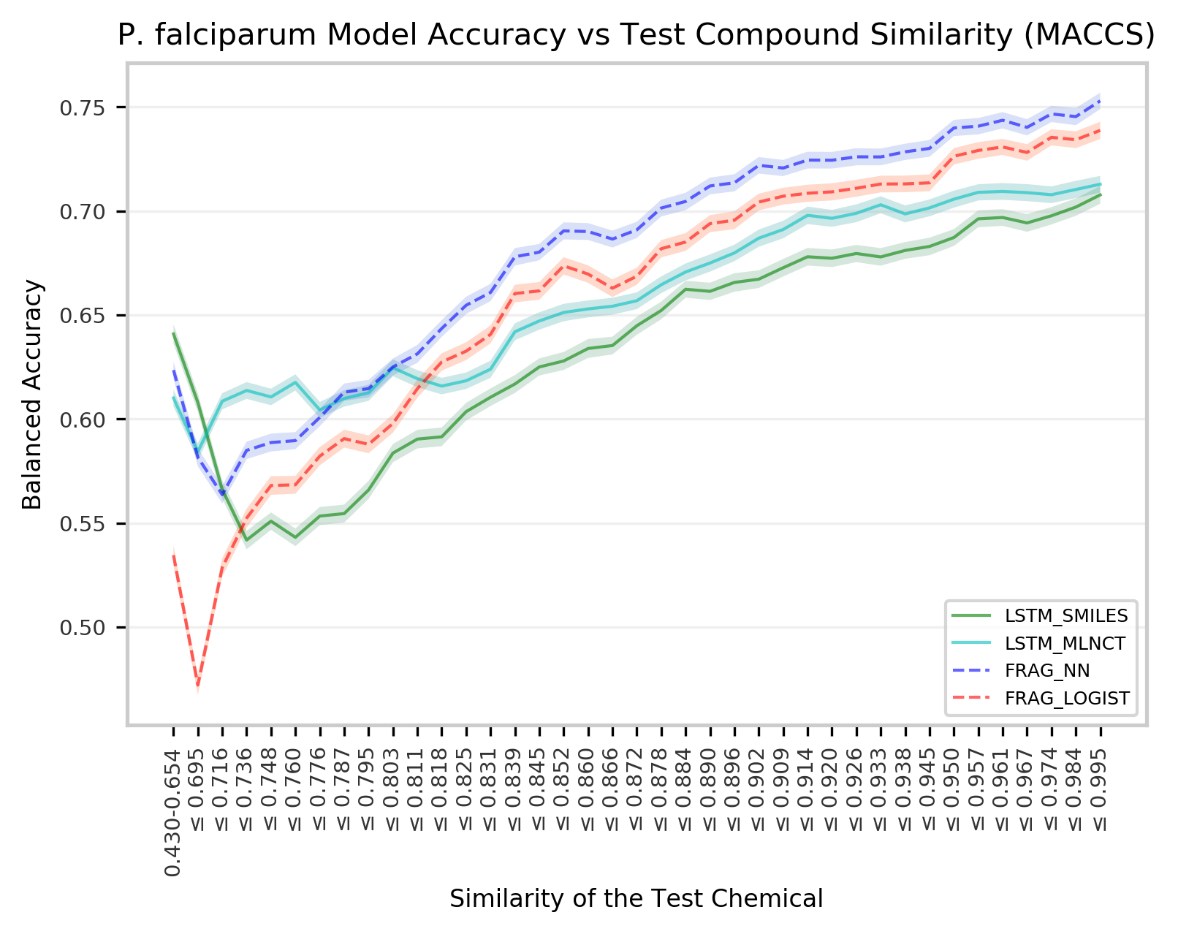


**Figure S12.** MACCS key similarity-based performance of the *P. falciparum* models for 1966 external set compounds the 7866 training set chemicals. Each step in the horizontal axis is composed of 50 test compounds. The confidence interval band around the lines were obtained using a bootstrap sampling process.

**Table S6.** Accuracy of different models with and without domain of applicability.

| Data set | Model | Test set *BAL_ACC* **without** domain of applicability | Test set accuracy **with** domain of applicability | |
| --- | --- | --- | --- | --- |
|  |  |  | *BAL_ACC* | Coverage |
| Ames Mutagenicity | LSTM_SMILES | 0.879 | 0.889 | 0.975 |
|  | LSTM_MLNCT | 0.867 | 0.878 | 0.967 |
|  | FRAG_NN | 0.875 | 0.887 | 0.963 |
|  | FRAG_LOGIST | 0.878 | 0.886 | 0.965 |
| Inhibition of Hepatitis C Virus (HCV) | LSTM_SMILES | 0.770 | 0.787 | 0.916 |
|  | LSTM_MLNCT | 0.769 | 0.795 | 0.900 |
|  | FRAG_NN | 0.790 | 0.811 | 0.919 |
|  | FRAG_LOGIST | 0.780 | 0.816 | 0.897 |
| Inhibition of *P. falciparum* Dd2 | LSTM_SMILES | 0.707 | 0.723 | 0.935 |
|  | LSTM_MLNCT | 0.715 | 0.737 | 0.904 |
|  | FRAG_NN | 0.752 | 0.768 | 0.928 |
|  | FRAG_LOGIST | 0.741 | 0.779 | 0.871 |
